# Cellular Response to Unfolded Proteins in Depression

**DOI:** 10.3390/life11121376

**Published:** 2021-12-10

**Authors:** Mateusz Kowalczyk, Edward Kowalczyk, Paweł Kwiatkowski, Łukasz Łopusiewicz, Monika Talarowska, Monika Sienkiewicz

**Affiliations:** 1 Babinski Memorial Hospital, Aleksandrowska St. 159, 91-229 Lodz, Poland; mateuszjerzykowalczyk@gmail.com; 2 Department of Pharmacology and Toxicology, Medical University of Lodz, Żeligowskiego St. 7/9, 90-752 Lodz, Poland; edward.kowalczyk@umed.lodz.pl; 3 Department of Diagnostic Immunology, Pomeranian Medical University in Szczecin, Powstańców Wielkopolskich 72, 70-111 Szczecin, Poland; pawel.kwiatkowski@pum.edu.pl; 4 Center of Bioimmobilisation and Innovative Packaging Materials, Faculty of Food Sciences and Fisheries, West Pomeranian University of Technology Szczecin, Janickiego 35, 71-270 Szczecin, Poland; lukasz.lopusiewicz@zut.edu.pl; 5 Department of Clinical Psychology and Psychopathology, Institute of Psychology, University of Lodz, Smugowa St. 10/12, 91-433 Lodz, Poland; monika.talarowska@now.uni.lodz.pl; 6 Department of Pharmaceutical Microbiology and Microbiological Diagnostic, Medical University of Lodz, Muszyńskiego St. 1, 90-151 Lodz, Poland

**Keywords:** endoplasmic reticulum stress, unfolded protein response, inflammation, depression

## Abstract

Despite many scientific studies on depression, there is no clear conception explaining the causes and mechanisms of depression development. Research conducted in recent years has shown that there is a strong relationship between depression and the endoplasmic reticulum (ER) stress. In order to restore ER homeostasis, the adaptive unfolded protein response (UPR) mechanism is activated. Research suggests that ER stress response pathways are continuously activated in patients with major depressive disorders (MDD). Therefore, it seems that the recommended drugs should reduce ER stress. A search is currently underway for drugs that will be both effective in reducing ER stress and relieving symptoms of depression.

## 1. Introduction

Depression (Latin depressio-depression, oppression) is a psychiatric disorder characterized by a pathologically low mood (hypothimia) and a negative attitude towards oneself, one’s status in the real world, and one’s future [1]. It is a complex and heterogeneous disease whose etiopathogenesis depends on many factors [2]. Despite the great medical and social importance of depressive disorder (DD) and many studies carried out on this entity, there is no clear conception explaining the causes and mechanisms of the development of this disorder. Several theories explaining the onset of depression were proposed that were confirmed by biochemical, immunological, and physiological studies. Parallel to the well-known "monoamine", "cytokine", "stress", and hypothalamic-pituitary-adrenal (HPA) axis theories of depression, the new theories, including altered neural plasticity of the brain, neurogenesis, and diurnal rhythm desynchronosis (chronobiological model), were suggested to explain the occurrence of depression [3,4,5,6].

Several preclinical and clinical studies have been performed recently, which show a strong relationship between depression and changes in the endoplasmic reticulum (ER) [7,8].

## 2. Endoplasmic Reticulum (ER)

This ER is the cell’s central organelle, which controls its metabolism and, depending on the needs, participates in protein or lipid synthesis [9,10]. ER is the site of protein folding, post-translational modification, and the center of directing membrane and secretory proteins to the appropriate areas of the cell. It is responsible for the maintenance of redox homeostasis, regulation of the cellular level of Ca^2+^ ions, and the course of cellular signaling pathways [10]. ER chaperones such as glucose-regulated proteins GRP78 and GRP94 are responsible for controlling the ER proper operation [11]. Numerous cellular processes require the involvement of ER chaperones. The transfer of the newly synthesized peptide to the ER membrane, protein folding, misfolded protein removal (by the ubiquitin-proteasome system or the autophagy-lysosome system), and the maintaining of calcium homeostasis have been proved as their functions [12,13,14,15]. The inability to adapt the course of the processes taking place in the ER to the organism’s needs or their impairment is referred to as ER stress. One of the main consequences of disturbing ER homeostasis is the impairment of protein folding processes, leading to the accumulation of unfolded proteins in this organelle [10].

Among the causes of the endoplasmic reticulum stress, inflammation is mentioned, which, apart from infection, hypoxia, metabolic disorders, and neurodegenerative diseases, often leads to the disruption of the ER function [16]. In order to restore ER homeostasis, the adaptive unfolded protein response (UPR) mechanism is activated [17,18]. The main task of the UPR is to remove defective, unfolded proteins by intensifying their folding processes, inhibiting the synthesis of new proteins and increasing the capacity of this organelle. The UPR has a second, equally important function of inducing cell apoptosis when the ER cannot be restored to equilibrium [10].

In mammals, three transmembrane proteins are the major ER stress receptors: ATF6 (activating transcription factor 6, gene ATF6), IRE1α (inositol-requiring ER-to-nucleus signaling protein, gene ERN1), and PERK (protein kinase-like endoplasmic reticulum kinase, gene EIF2AK3) [19].

Under homeostatic conditions, these three proteins are associated with the chaperone protein. BiP (binding protein) is a protein found in the light of the reticulum and is also known as GRP78. BiP prevents the activation of the above-mentioned enzymes. Under homeostatic conditions, GRPs are responsible for chaperone, Ca2 + binding, and cytoprotection [20]. Each GRP can also be assigned specific properties.

GRP94 is responsible for looking after Toll-like receptors, which play a key role in the function of antigen presenting cells (APCs) that produce pro-inflammatory cytokines and stimulate an adaptive immune response [21]. Thus, increased expression of GRP94 triggers inflammatory responses in cells [22,23].

Under conditions of the reticulum stress, BiP as a chaperone binds to misfolded proteins, releasing the transmembrane proteins IRE1, PERK, and ATF6. Once released, IRE1 undergoes homodimerization and autophosphorylation. This allows IRE1 to activate as an RNAse, enabling the excision of the 26-nucleotide intron from the mRNA for the XBP1 protein (X-box DNA binding protein). The XBP1 mRNA encodes a transcription factor, and its cleavage by IRE1 enables the formation of a mature mRNA and subsequent translation of XBP1 [24]. Active XBP1 leaves the ER and is translocated to the nucleus, where it begins transcriptional activity.

The XBP1 protein binds to the regions of ERSE (ER stress element) within DNA [CCAAT (N9) CCACG] present in the promoters of many genes of the UPR pathway, activating, among others, transcription of genes for the heat shock protein family and other reticulated chaperones, as well as XBP1 itself [25]. In addition, XBP-1 has been found to bind constitutively to genes involved in ER homeostasis, which includes processes, such as the involvement in ER membrane biosynthesis, disulfide bond formation, protein folding, and protein translocation [26]. XBP-1 is also necessary for the activity of cytokines, such as TNF-α [27], and is considered an important factor in determining whether a cell will follow a pro-apoptotic pathway or be saved from the ER stress [24].

The PERK protein kinase dimerizes when released from association with BiP and, similarly to IRE1, it undergoes trans-autophosphorylation. Active PERK phosphorylates the subunit of the second eukaryotic translation initiation factor 2 (eIF2a) [23]. The phosphorylated form of eIF2a has a lower recognition of the AUG translation initiation codon, which causes the inhibition of protein synthesis depending on the presence of the cap, i.e., 7-methylguanosine [22]. Such translation control helps to reduce the amount of misfolded proteins in the cell undergoing stress from the endoplasmic reticulum and allows it to survive.

Molecules having special regulatory sequences called IRES (internal ribosome entry site) can bypass the PERK-dependent translation block. An example of such a molecule is the activating transcription factor 4 (ATF4) [22,23]. It has been shown that ATF4 can affect cell survival by inducing genes related to amino acid metabolism or changing the redox potential [28]. On the other hand, ATF4 is recognized as the strongest signal for the transcription of the pro-apoptotic factor CHOP (CEBP homologous protein) [29]. The transcription factor ATF6 is another receptor activated by misfolded proteins. After detaching from the BiP protein, it enters the Golgi apparatus, where is activated by cleavage by S1P (site 1 protease as the first one to cut the SREBP transcription factor) and S2P (site 2 protease as the second one to cut SREBP). The resulting protein has a leucine zipper structure which, like XBP1, binds to ERSE regions within DNA, but only in combination with a transcription factor CBF (CCAAT binding factor). ATF6 increases the transcription of XBP1, BiP, calreticulin, protein disulfide isomerase (PDI), and CHOP [22,23,24].

If the efforts to rebalance the ER turn out to be insufficient, the UPR initiates cell apoptosis. IRE1 and PERK are involved in the activation of the pro-apoptotic JNK (c-JunNH2-terminal kinase), and ATF4 is responsible for the activation of the pro-apoptotic transcription factor CHOP (growth arrest and DNA-damage-inducible protein 153, GADD153 gene) [30,31].

The PERK-ATF4-CHOP pathway can induce apoptosis by interacting with death receptors 4 (DR4) and DR5. CHOP increases the DR5 expression by binding to the 5′ region of the DR5 gene [32]. If the stress of the endoplasmic reticulum is irreversible, the PERK-ATF4-CHOP pathway will allow DR5 mRNA to grow and accumulate in the ER.

## 3. Research on the Role of ER in Depression

In recent years, several clinical and preclinical studies have been carried out that show that the ER stress may be responsible for the occurrence of depression. For example, there is a strong relationship between depression and the UPR [33,34]. UPR is known as an inducer of cytokines and inflammation. It has been found that the increase in the level of IL-6, TNF-α, IL-1β, and their soluble receptors is mediated by the UPR in patients with severe depression [35]. CHOP, one of the key UPR regulators, directly induces caspase 11, which activates IL-1β. The UPR also acts on other pro-inflammatory cytokines, such as IL-8 [36] and TNF-α [27] via the IRE-1-XBP-1 and IL-23 pathways [37]. The above-described connections of ER stress with inflammation are important because one of the theories of depression is the theory of inflammation. Many experimental studies have been described that show a significant increase in the concentration of pro-inflammatory cytokines in the blood, cerebrospinal fluid, and in many brain centers in patients with depression and in an experimental model of depression in animals [38,39]. According to the literature, the relationship between depression and inflammation is indicated by the following relationships: in patients with inflammatory diseases, the frequency of depression is higher, about 1/3 of patients with depression have increased levels of inflammatory biomarkers, and patients treated with cytokines are at an increased risk of depression [40]. Other authors emphasize that environmental stressors, especially traumatic events in early life, are one of the strongest depression risk factors described so far [41]. Long-term mental stress can disrupt the functions of the hippocampus and nerve regeneration, inhibit the action of dopamine, and change the sensitivity of the amygdala to negative stimuli [42]. 

**Figure 1:**
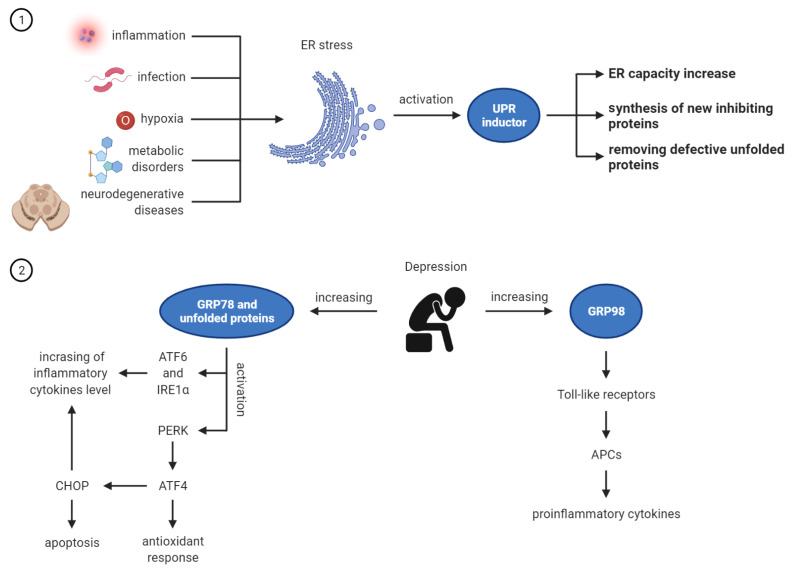
ER stress and depression: UPR activation under the influence of stressors and its role in ER homeostasis restoring; Increased expression of GRP78 and GRP94 in depression. Made in ©BioRender—biorender.com (accessed on 1 September 2021).

Relationship between ER stress and depression is presented in the Figure 1.

**Figure 2:**
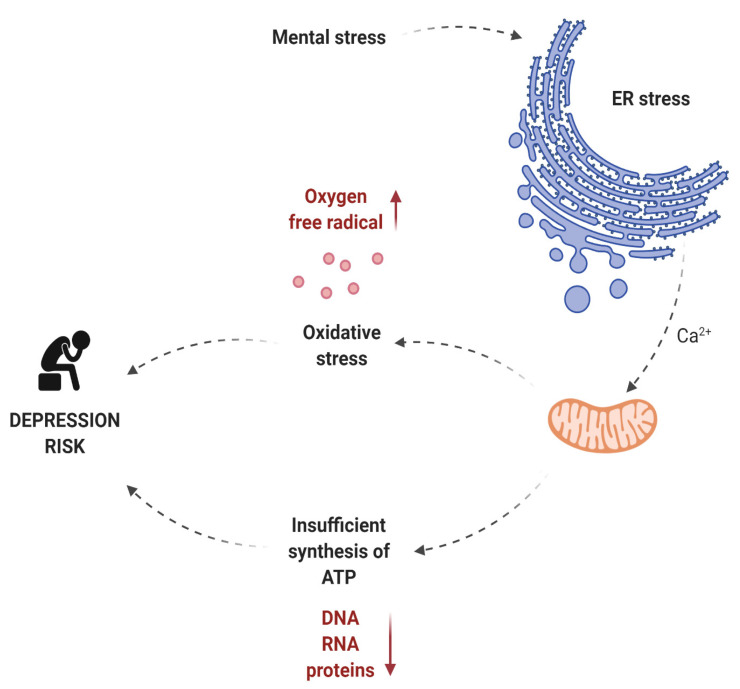
The role of ER stress and mitochondria in the development of depression. Made in ©BioRender—biorender.com (accessed on 4 December 2021).

Liu et al. noted that mental stress causes oxidative stress, ER stress, and insufficient ATP (adenosine triphosphate) synthesis [43]. ATP is produced in the mitochondria and is the primary source of energy for most cellular functions. The energy released during ATP hydrolysis is used for the synthesis of macromolecules, DNA, RNA, and proteins and for the transport of ions and macromolecules across the cell membrane. ATP is also necessary in the process of signal transmission in the cell and is used by kinases as a source of phosphate groups [44]. Given that neurons are highly specialized cells whose main role is to generate potential differences and transmit electrical impulses to other cells, it is not surprising that they use a lot of energy. The dependence of neurons on constant high ATP production (in the mitochondria) is their weak point. The inability to accumulate energy reserves in the form of glycogen, to switch metabolism from oxidative phosphorylation to glycolysis, or to use substrates other than oxygen as the final electron acceptor, is the reason why these cells are sensitive to the lack of oxygen and glucose [44]. The participation of mitochondria in the development of depression is associated not only with the reduction in the ATP synthesis. Mitochondria also participates in the formation of oxygen free radicals, and thus participates in oxidative stress (which is one of the many risk factors for depression). Perhaps the involvement of mitochondria in oxidative stress or reduced energy production is a consequence of exposure of mitochondria to ER stress. According to Bravo et al., ER stress can lead to mitochondrial damage and induction of oxidative stress. During ER stress, calcium cations released from the ER light can be captured by nearby mitochondria, causing damage to the mitochondria and thus increasing oxygen free radical production and pro-apoptotic signaling [45]. IRE1α interacts with Bak and Bax (pro-apoptotic members of the Bcl-2 family) and increases mitochondrial dependent cell death. Moreover, both the mitochondria and the ER are physically and functionally connected by the membranes of the ER associated with the mitochondria. Recent studies have shown that the ER protein folding process is strongly related to the production of oxygen free radicals [46,47,48]. The involvement of ER stress and mitochondria in the development of depression is presented in Figure 2.

Jangra et al. [49] in a study in a mouse model of depression, showed that the levels of GRP78 and CHOP expression in the hippocampus and CHOP in the prefrontal cortex, were significantly increased in the study group. Sodium phenylbutyrate (ER stress inhibitor) and edaravone (free radical scavenger) reduce the expression of these genes and also counteract the persistent symptoms of depression in this model of depression. Liu et al. found that not only the GRP78, but also XBP1 expression was significantly increased in the hippocampus in C57BL/6J mice [50].

Activation of the PERK-eIF2α signaling pathway in the hippocampus, thus lowering the level of expression of the brain-derived neurotrophic factor, and disturbances in behavior and memory similar to depression may occur under the influence of chronic stress [51]. The effect on improving memory in mice by inhibiting PERK expression in the hippocampus was shown by Sharma et al. [52]. This proves that cognitive functions depend on the PERK expression level. In addition, the increase in plasma corticosterone levels and expression of genes encoding GRP78, GRP94, ATF6, XBP1, ATF4, and CHOP of rats with learned helplessness was confirmed, which indicates depressive behavior. Hypercortisolemia is known to cause depression [53]. In people suffering from depression, an increased concentration of glucocorticoids not only in plasma, but also in urine and cerebrospinal fluid is observed [54]. In a significant proportion of depressed patients, besides hypercortisolemia, an altered rhythm of this hormone secretion is found, and in about 50% of patients, no inhibition of its secretion after administration of dexamethasone can be noted [5]. The hypothalamic-pituitary-adrenal axis is modulated by a feedback loop, which normally causes high levels of corticosteroids (CS) to bind to adequate receptors in the hypothalamus and pituitary gland, blocking their further stimulation [55]. This mechanism fails in the case of depression, and cytokines increase the generated CRH (corticotropin-releasing hormone) release of ACTH (adrenocorticotropic hormone) and cortisol. This is probably due to a reduction in the number/sensitivity of GR (glucocorticoid receptor) receptors located in the limbic system, hypothalamus, and pituitary gland [56]. Corticosteroids have a strong negative effect not only on the hippocampus and amygdala, but also on the functioning of the prefrontal cortex [57].

According to research studies, survival and regeneration of neurons depend on the chronically activated ER stress in the brain [58]. Long-term stress in the course of depressive episodes can disrupt the functions of the hippocampus, nerve regeneration, inhibit the action of dopamine, and change the sensitivity of the amygdala to negative stimuli [42].

The relationship between ER stress and its effect on the pathophysiology of depression was investigated by Behnke et al. [12] Higher expression levels of GRP78, GRP94, and calreticulin in a post-mortem examination were found in the temporal cortex of MDD patients with major depressive disorders who died of suicide compared to those of MMD patients after non-suicidal deaths. Nevell et al., after analyzing the levels of GRP78, CHOP, and XBP1 expression in leukocytes in MDD patients, noticed that MDD patients had significantly higher levels of GRP78, CHOP, and XBP1 compared to the control group [59].

## 4. Conclusions

Perhaps, a detailed understanding of the changes taking place in the ER in depression will allow us to understand the mechanism of its formation and lead to the development of an appropriate therapeutic procedure to slow down or stop this disease. The current guidelines for the treatment of depression suggest selective serotonin reuptake inhibitors (SSRIs), serotonin norepinephrine reuptake inhibitors (SNRIs), and other drugs, including agomelatine, bupropion, mirtazapine, and vortioxetine as first-line treatment [60,61].

The recommendations for the use of these drugs were dictated by the results of previous studies on the causes of depression. Many patients are unable to benefit from the long-term advantages of the available antidepressants and adjuvants as these drugs lose their effectiveness or have unbearable side effects. Side effects of some medications include weight gain, elevated blood sugar, and cholesterol levels resulting in an increased risk of diabetes, high blood pressure, and cardiovascular disease [62]. It is also known that the side effects of antidepressants may cause changes in the endoplasmic reticulum, as reticulum stress may be caused, inter alia, by metabolic disorders. It is impossible to predict which drugs will elicit a positive response in any particular patient, and some patients do not respond to therapies that target all known specific neurotransmitters. It can be assumed that one of three patients with depression is "resistant to treatment" [63]. Perhaps, the lack of effect of antidepressants is related to their influence on the endoplasmic reticulum and induction of its stress.

Scientific studies conducted in animals show that sertraline used in depression may have adverse effects on the brain. Sertraline belongs to the SSRI group and is approved by the Food and Drug Administration (FDA) for PTSD (Post-traumatic stress disorder). This drug is also assumed to act as an inhibitor of the Sig1R receptor, which is expressed on ER membranes. Several functions have been attributed to Sig-1R, including the regulation of ion channels such as the Ca^2+^ and K^+^ channel, inhibition of Ca^2+^ influx by the N-methyl-D-aspartate (NMDA) receptor, modulation of the release of neurotransmitters, such as dopamine, regulation of lipid distribution, and cell differentiation [64]. Sig-1R has also been shown to have neuroprotective effects and several studies have reported that it acts as a molecular chaperone [64,65,66,67]. Under normal conditions, the Sig-1R forms a complex with another caring molecule GRP78 BiP of the ER membrane. Under stress, ER Sig-1R dissociates from BiP, interacts with IP3 receptors and stabilizes the structure of the IP3 receptor [67]. Research on the role of Sig-1R in the pathogenesis of mental illness has indicated that Sig-1R expression is reduced in patients with schizophrenia [68]. Moreover, the Sig-1R knockout mice show symptoms of depression [69]. When a cell encounters ER stress, the Sig-1R expression increases in response to activation of the PERK pathway, which is one of the cellular responses to ER stress. In addition, the induction of Sig-1R expression suppresses cell death signals induced by ER stress [70]. So, the authors began to study drugs for their effect on Sig-1R. It has been noted that repeated administration of phencyclidine (PCP) (10 mg/kg/day, 10 days) in mice is associated with lower Sig1R protein densities in the frontal cortex and hippocampus, as well as with cognitive deficits. This deficit cannot be reversed by administering sertraline, but can be done by administering fluvoxamine (a known SSRI antidepressant). In the study of resistance to leptin, Hosoi et al. disclosed that fluvoxamine, a selective serotonin reuptake inhibitor (SSRI) with high affinity for the σ-1 receptor (Sig-1R), weakened ER stress by Sig-1R [71,72]. The study by Omi et al. showed that fluvoxamine-mediated upregulation of Sig-1R promoted neuroprotection by inhibiting ER-induced apoptosis [73]. Ma et al. in turn showed that fluoxetine induced apoptosis in glioblastoma cells through ER stress-related CHOP apoptotic pathways, such as PERK/eIF2α/ATF4/CHOP and ATF6/CHOP signaling pathways [74].

Desipramine promotes antitumor activity in glioblastoma by inducing autophagy through the PERK/eIF2α and ATF6 signaling pathways [75], and the rapid antidepressant effect of ketamine may also be associated with ER stress [76]. Several studies suggest that ketamine, through glutamate and/or neurotrophic receptors, stimulates the mTOR pathway in the prefrontal cortex (PFC) [77,78]. This is important because research indicates a relationship between synaptic protein deficits and mTOR signaling dysregulation in MDD [79]. Post-mortem studies have shown significant deficits in the mTOR signaling in PFC in people diagnosed with MDD [80]. Rapid activation of the mTOR pathway results in a rapid increase in synapse-related proteins and induces the mechanism for the rapid antidepressant effect of the NMDAR antagonist ketamine [67]. mTOR regulates protein synthesis by phosphorylation and inactivation of the mRNA translation repressor, 4E-BP1 [81]. Cellular processes including apoptosis, autophagy, translation, energy metabolism, and inflammation are controlled by mTOR kinase and the endoplasmic reticulum (ER) stress pathway [82]. Kato et al. [83] and Nakajima et al. [84] indicated that in some pathological situations the cellular toxicity caused by ER stress is associated with chronic activation of the mTOR 1 complex (mTORC1).

Abelaira et al. [76] demonstrated that in the group of animals receiving ketamine in combination with rapamycin, there was a decrease in the levels of PERK and IRE1-alpha in the PFC, suggesting that rapamycin was able to block the effect of ketamine on these parameters. According to Wang et al., sustained mTORC1 activation initiates protein synthesis and UPR activation, while in the later phase it induces ER stress [85].

In conclusion, it should be emphasized that the knowledge of the influence of antidepressants on the ER is important in the context of their antidepressant activity. Many experimental and clinical studies confirm changes in ER in depression.

## 5. Further Research Directions

Once the relationship between ER stress and depression was established, a search began for drugs that would be both effective in reducing ER stress and relieving symptoms of depression. Therefore, the existing antidepressants were analyzed in terms of their influence on ER stress biomarkers. It turns out that there are several drugs that can worsen ER stress. The search for the perfect drug is still ongoing. One of the lines of research is the Bax 1 (BI-1) inhibitor. Bax is an evolutionarily conserved ER protein that has an antiapoptotic effect on the ER and inhibits stress-induced cell death [86]. Sui et al. [87] believe that BI-1 may play a protective role in depression caused by chronic mild stress. The results of Hunsberger et al. [88] further indicate that BI-1 protects against anhedonia. In terms of modern psychopathology, anhedonia most often means a decrease in interest or the ability to experience pleasure and/or joy in response to stimuli that usually caused this positive reaction in a given person [89,90]. Anhedonia can be an element of the clinical picture of many mental and neurological disorders, but most often and in the most typical way, it occurs in the course of a depressive episode, as one of its axial symptoms. Scientific evidence also suggests that an estrogen β agonist can alleviate ER stress-induced anxiety and depression by preserving the IRE1α/XBP1 pathway [91]. Finally, a soluble epoxide hydrolase inhibitor has been found in rats to ameliorate the development of depressive behavior by alleviating ER stress [92]. There is an increasing list of UPR inhibitors and activators [93]; however, none of them have been routinely used in clinical trials for depression. 4-Phenylbutyrate (4-PBA), approved for the treatment of urea cycle disorders, and TUDCA for primary cholangitis are chemical chaperones designed to aid in protein folding and thus reduce ER stress, although their exact action is not understandable [94,95].

Clinical trials are ongoing on the systemic use of ER stress-reducing drugs in several diseases, including diabetes and obesity [96]. 4-Phenylbutyrate (4-PBA) has been shown to contribute to the treatment of spinal muscular atrophy by altering gene expression patterns [97,98]. Moreover, 4-PBA is able to inhibit disease progression and neuritis in multiple sclerosis [99]. Studies suggest that 4-PBA is also involved in inhibiting ER stress-induced ischemic damage through the transcriptional regulation of CHOP and GRP78 [100,101,102]. 

A promising compound seems to be trazodone, which is a serotonin reuptake inhibitor and an antagonist of 5-HT2 receptors. A beneficial therapeutic effect may occur after about a week of treatment. It reduces psychomotor inhibitions, has a positive effect on the mood, has an anti-anxiety effect, and restores the physiological rhythm of sleep. Trazodone lowers ATF4 levels without affecting eIF2α in cell cultures that have been treated with tunicamycin (ER stress activator) [103]. The role of ER stress in depression, and at the same time the influence of antidepressants on the elements of the endoplasmic reticulum are therefore an interesting direction of research. Doctors and depressed patients are still waiting for a drug that will reduce the number of pharmacotherapy failures of this disease entity.
